# Chronic hypersensitivity pneumonitis: identification of key prognostic determinants using automated CT analysis

**DOI:** 10.1186/s12890-017-0418-2

**Published:** 2017-05-04

**Authors:** Joseph Jacob, Brian J. Bartholmai, Ryoko Egashira, Anne Laure Brun, Srinivasan Rajagopalan, Ronald Karwoski, Maria Kokosi, David M. Hansell, Athol U. Wells

**Affiliations:** 1 Department of Radiology, Royal Brompton Hospital, Royal Brompton and Harefield NHS Foundation Trust, London, UK; 2 Division of Radiology, Mayo Clinic Rochester, Rochester, MN USA; 3 Department of Radiology, Faculty of Medicine, Saga University, 5-1-1 Nabeshima, Saga-City, Japan; 4 Department of Physiology and Biomedical Engineering, Mayo Clinic Rochester, Rochester, MN USA; 5 Interstitial Lung Disease Unit, Royal Brompton Hospital, Royal Brompton and Harefield NHS Foundation Trust, London, UK

**Keywords:** Chronic hypersensitivity pneumonitis, Pulmonary vessel volume, Idiopathic pulmonary fibrosis, Quantitative CT analysis

## Abstract

**Background:**

Chronic hypersensitivity pneumonitis (CHP) has a variable disease course. Computer analysis of CT features was used to identify a subset of CHP patients with an outcome similar to patients with idiopathic pulmonary fibrosis (IPF).

**Methods:**

Consecutive patients with a multi-disciplinary team diagnosis of CHP (*n* = 116) had pulmonary function tests (FEV1, FVC, DLco, Kco, and a composite physiologic index [CPI]) and CT variables predictive of mortality evaluated by analysing visual and computer-based (CALIPER) parenchymal features: total interstitial lung disease (ILD) extent, honeycombing, reticular pattern, ground glass opacities, pulmonary vessel volume (PVV), emphysema, and traction bronchiectasis. Mean survival was compared between both CHP and IPF patients (*n* = 185).

**Results:**

In CHP, visual/CALIPER measures of reticular pattern, honeycombing, visual traction bronchiectasis, and CALIPER ILD extent were predictive of mortality (*p* < 0 · 05) on univariate analysis. PVV was strongly predictive of mortality on univariate (*p* < 0 · 0001) and multivariate analysis independent of age, gender and disease severity (represented by the CPI [*p* < 0 · 01]). CHP patients with a PVV threshold >6 · 5% of the lung had a mean survival (35 · 3 ± 6 · 1 months; *n* = 20/116 [17%]) and rate of disease progression that closely matched IPF patients (38 · 4 ± 2 · 2 months; *n* = 185).

**Conclusions:**

Pulmonary vessel volume can identify CHP patients at risk of aggressive disease and a poor IPF-like prognosis.

**Electronic supplementary material:**

The online version of this article (doi:10.1186/s12890-017-0418-2) contains supplementary material, which is available to authorized users.

## Background

The majority of patients with hypersensitivity pneumonitis who present to specialist centres have the chronic fibrotic form of the disease [1–3]. Within the population of chronic hypersensitivity pneumonitis (CHP) patients, it has been observed that some patients may show an accelerated rate of progression, comparable to the trajectory of idiopathic pulmonary fibrosis (IPF) [2, 4, 5]. The overlap with IPF is also evident in the often-challenging differentiation of CHP from IPF in a multi-disciplinary team (MDT) setting [5–7]. The early identification of a rapidly progressive fibrosing lung disease is increasingly relevant in an era of anti-fibrotic therapies, in which treatment guidelines are likely to increasingly broaden the spectrum of diagnoses for which anti-fibrotic treatment is indicated.

Given the varied outcome and unpredictable prognosis in CHP, the American National Heart, Lung and Blood Institute, in collaboration with the Office of Rare Diseases, convened a workshop in 2005 to discuss future research priorities in HP [8]. Amongst the various recommendations was a need to improve the characterisation of different HP phenotypes. The workshop also emphasized the desirability of exploring quantitative CT analysis in longitudinal studies of HP patients [8].

Automated computer-based quantitative imaging has advanced rapidly in recent years, with quantitatively scored CT variables shown to be superior to visual CT scores in predicting mortality in IPF. [9] To date however, quantitative CT tools have not been applied to the study of outcome in patients with CHP. Our study specifically set out to identify whether, in patients without end-stage lung disease, there are visual or quantitative CT features that characterize CHP patients who have a poor IPF-like outcome.

## Methods

### Study population and clinical information

**Fig. 1:**
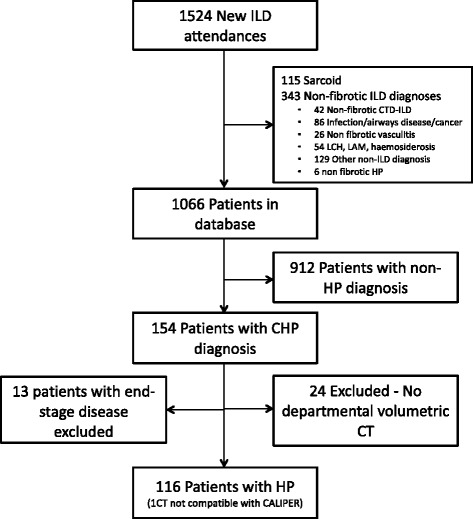
Flowchart illustrating the selection of patients for the final chronic hypersensitivity pneumonitis study population. ILD = interstitial lung disease, CTD = connective tissue disease, CHP = chronic hypersensitivity pneumonitis, LCH = Langerhans cell histiocytosis, LAM = lymphangioleiomyomatosis, CT = computed tomography

All new consecutive patients with an MDT diagnosis of CHP and IPF according to published guidelines [10–12], over a four and a half year period (January 2007–July 2011) were identified from the Interstital Lung Disease Unit database. Patients with a non-contrast, supine, volumetric thin-section CT were captured. Individuals with no overt CT signs of fibrosis (the absence of honeycombing/reticular pattern/traction bronchiectasis on visual scoring by both radiologist scorers, RE and ALB) were excluded from analysis (Fig. 1).

Approval for this retrospective study of clinically indicated CT and pulmonary function test (PFT) data was obtained from the Institutional Ethics Committee and informed patient consent was not required.

CT scanning, echocardiography, PFT protocols were as previously described [9, 13] and are included in the online appendices. CALIPER CT data processing steps [9, 13] are also detailed in the online appendices.

### CALIPER CT pattern quantitation

**Fig. 2:**
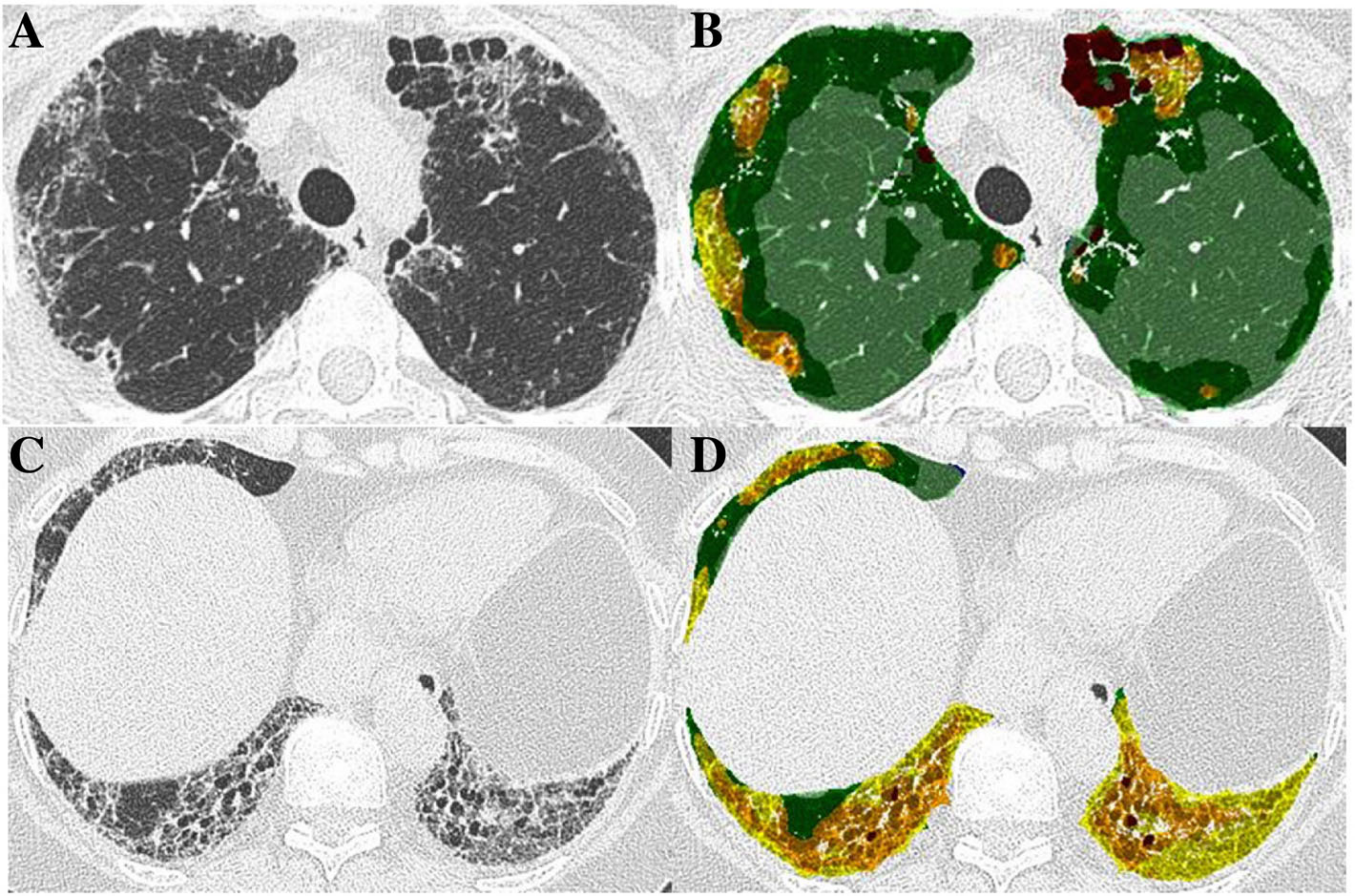
Axial CT image in a 46-year-old female never smoker (**a**), diagnosed with chronic hypersensitivity pneumonitis following surgical lung biopsy. An upper lobe predominant fibrosing lung disease associated with honeycomb cysts is demonstrated. On the CALIPER colour overlay image (**b**), ground glass opacities (yellow) are interspaced with reticular pattern (orange) in the lung periphery. In the anterior left upper lobe, there are honeycomb cysts visible (brown). Light and dark green areas represent normal lung parenchyma. Axial CT image in a 55-year-old female 2-pack-year ex-smoker (**c**), diagnosed with chronic hypersensitivity pneumonitis following surgical lung biopsy with more extensive fibrosis involving the lower lobes. CALIPER colour overlay images show minor misclassification of traction bronchiectasis as honeycomb cysts in the left lower lobe (**d**), but extensive ground glass opacities (yellow) and interspaced reticular pattern (orange)

**Fig. 3:**
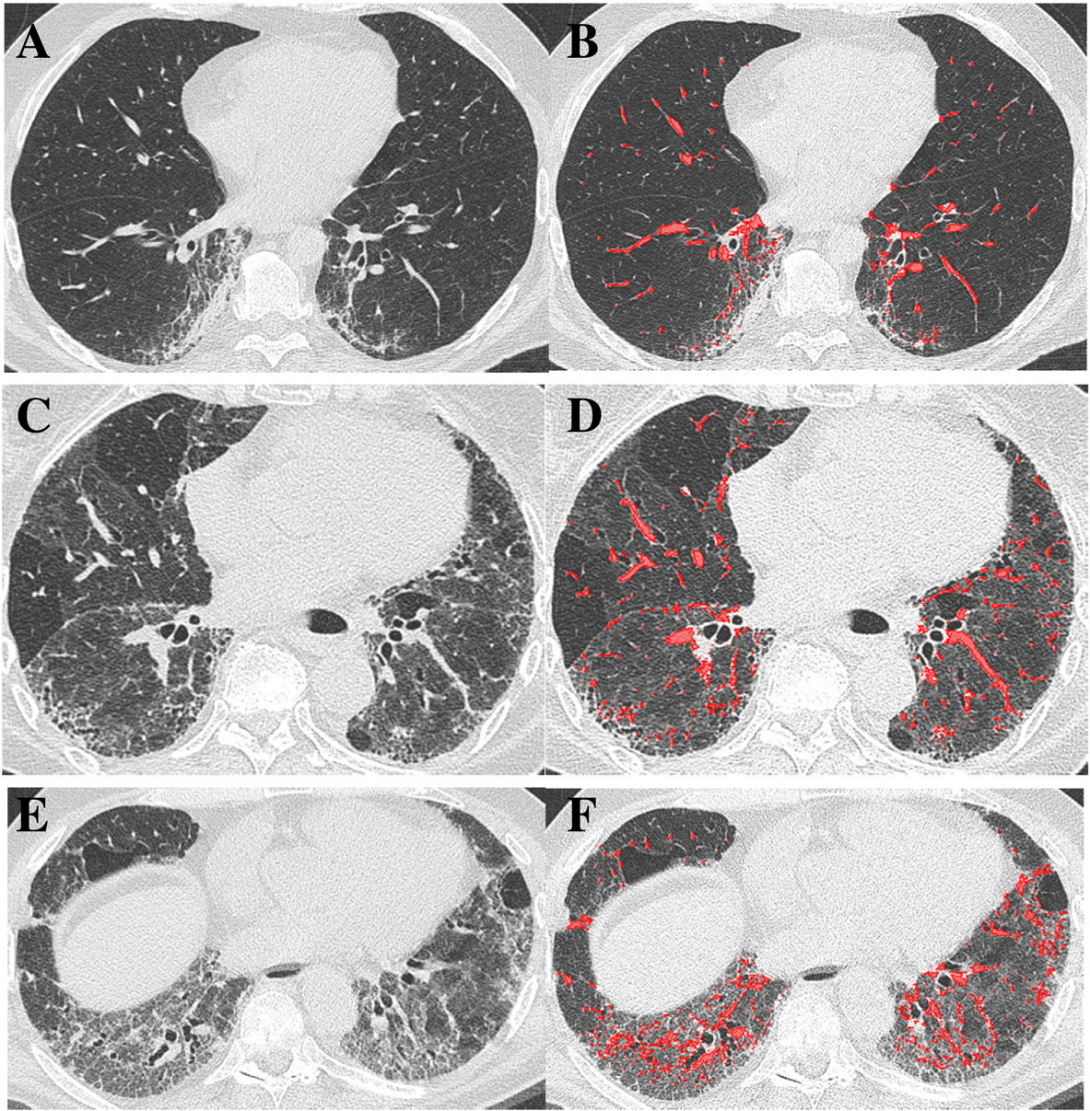
Corresponding axial CT images and colour overlay maps of CALIPER quantitation of pulmonary vessel volume (red), a volumetric measure of pulmonary arteries and veins within the lung, excluding vessels at the lung hilum in three chronic hypersensitivity pneumonitis patients with mild (**a** + **b**), moderate (**c** + **d**) and severe (**e** + **f**) extents of fibrosis. Note there is misclassification of small regions of the reticular pattern in the paravertebral regions (**b**) and right lower lobe (**f**) as vessels

CALIPER evaluation of CT data involved algorithmic identification and volumetric quantification of 15x15x15 voxel volume units into one of six radiological parenchymal features: ground glass opacity (GGO), reticular pattern, honeycombing, emphysema, pulmonary vessel volume (PVV), and normal lung (Figs. 2 and 3).

A *fibrosis score* represented the sum of reticular pattern and honeycombing whilst the *ILD extent* additionally summed GGO extent. PVV represented the sum of the volumes of the pulmonary arteries and veins, excluding vessels at the lung hilum. All parenchymal pattern volumes were divided by the total lung volume, also derived by CALIPER, to generate percentages for each pattern.

### CT visual quantitation

Each CHP CT was evaluated independently by two radiologists (ALB, RE with 5 and 7 years thoracic imaging experience respectively), blinded to all clinical information. Methods by which discordant CT scores were consensed are given in the Additional file 1.

Additional visually quantified CT features included consolidation, mosaicism (decreased attenuation component), and traction bronchiectasis (no comparable CALIPER score) [details in Additional file 1]. There was no analogous visual score of PVV. A CT index of pulmonary hypertension (main pulmonary artery:ascending aorta ratio) was recorded by a single scorer using electronic caliper measurements of the ascending aorta and pulmonary artery diameters at the level of the pulmonary artery bifurcation [14, 15]. A CT UIP pattern was evaluated by one radiologist (JJ, with 5 years thoracic imaging experience) who was blinded to all clinical information.

### Identification of CHP patients with end-stage disease

The study aim was to identify characteristics of the subset of CHP patients who do not have end-stage disease yet progress to death rapidly with an IPF-like trajectory. Two methods were used to identify CHP with end stage disease:

1. Contained within the consecutive CHP cases were individuals with respiratory failure secondary to end-stage fibrosing lung disease, which in itself predicates a poor outcome. Pa0_2_ values were examined to separate out patients who would have an inevitably rapid decline because of end-stage disease at baseline. 102/129 (79%) patients had a measured Pa0_2_. Patients were identified as having end-stage disease if they had a Pa0_2_ < 7 · 5kPa (*n* = 13). In the 27 patients without a measured Pa0_2_, the use of supplementary oxygen therapy (recommended at a threshold of <7 · 3kPa0_2_ [10]) was used to identify patients with respiratory failure and therefore end-stage disease (*n* = 3). The first study group consisted of 116 CHP patients.
2. A threshold of FVC ≥ 50% predicted was also used to separate CHP patients into those with and without end-stage disease at baseline. 98/123 (80%) patients had a FVC ≥ 50% predicted. In 6 patients without a measured FVC, only one patient required supplemental home oxygen and was therefore excluded. The second study group consisted of 103 CHP patients. Analysis of this second population of non end-stage CHP patients is provided in Additional files 1, Additional file 2 and 3.

### Statistical analysis

Data are given as medians, means with standard deviations, or numbers of patients with percentages where appropriate. Interobserver variation for visual scores was assessed using the single determination standard deviation. Univariate and multivariate Cox regression analyses of the CHP cohort were used to investigate relationships within and between the three data sets: CALIPER CT evaluation, visual CT evaluation and PFTs. To evaluate group differences, the *T*-test and Mann Whitney *U* test were used for mean and median continuous variables and the Chi-squared tests was used for categorical variables.

Cox regression analyses of CALIPER and PFT variables were used to compare the CHP and IPF cohorts. The robustness of the results was confirmed using bootstrapping and resampling of the dataset up to 1000 times. Survival curves were created using Kaplan Meier analyses and statistically significant differences between curves evaluated using the Log-Rank test. In all study analyses, a p-value of <0.05 was considered significant. Assumptions of linearity and proportional hazards were tested by visual inspection of Martingale residuals and scaled Schoenfeld residuals. Statistical analyses were performed with STATA (version 12, StatCorp, College Station, TX, USA).

## Results

### Demographic data

The initial study population, which has not been previously described, comprised 129 consecutive patients newly presenting with an MDT diagnosis of CHP based on a compatible clinical history and review of the following data: antigen exposure history (positive in 53/129 [41%] patients), precipitating antibodies (positive in 50/129 [39%] patients), bronchoalveolar lavage (BAL) findings (performed in 73/129 [57%] patients), CT imaging (129/129 [100%] of patients), and histopathology (60/129 [46%] of patients).

The median age at presentation was 60 years. Patient status and mean follow up time (58.0 ± 17 · 5 months) were obtained by contacting the patient’s primary care giver on a given date. 50/129 patients (39%) died during the study period. Data on vital status was completed on 98% of cases with 3 patients censored. 41/73 (56%) patients undergoing BAL had a lymphocytosis ≥20% and 25/73 (34%) had a lymphocytosis ≥30%. A right ventricular systolic pressure (RVSP) >50 mmHg measured on transthoracic echocardiography was considered representative of pulmonary hypertension [16], and was found in 10/69 (14%) of the CHP cohort.

**Fig. 4:**
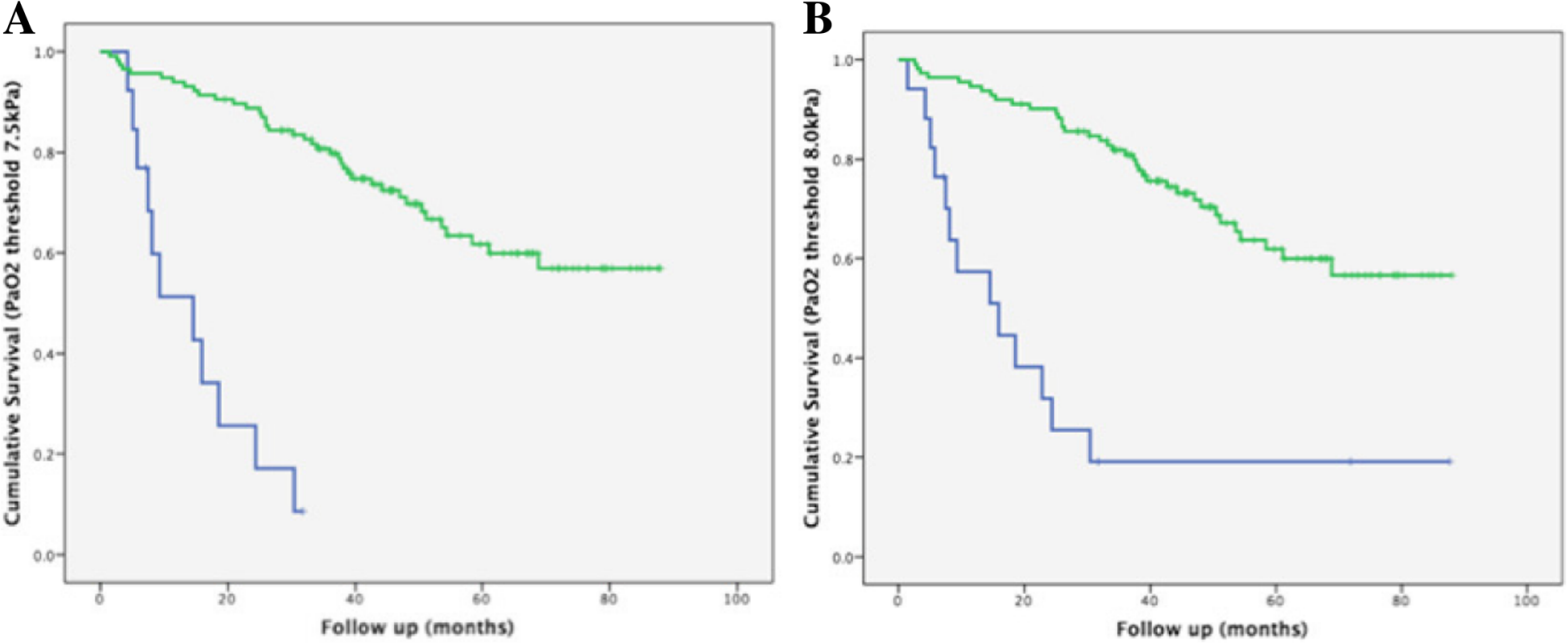
Survival curves demonstrating differences in survival between chronic hypersensitivity pneumonitis patients with differing PaO2 thresholds. **a** Differences between patients with a PaO2 >7 · 5kPa (green)(*n* = 116; mean survival = 65 · 6 ± 2 · 9 months) and patients with a PaO2 < 7 · 5kPa (blue)(*n* = 13; mean survival = 14 · 9 ± 2 · 7 months). Log rank test *p* = <0 · 0001. **b** Differences in survival between patients with a PaO2 > 8kPa (green)(*n* = 112; mean survival = 66 · 0 ± 2 · 9 months) and patients with a PaO2 < 8kPa (blue)(*n* = 17; mean survival = 27 · 4 ± 7 · 6 months). Log rank test *p* = <0 · 0001

When separating the CHP population into those with and without end-stage disease using a PaO_2_ threshold of 7 · 5kPa0_2,_ using Kaplan Meier curves, the 7 · 5kPa0_2_ threshold was shown to clearly distinguish groups with a good and bad outcome (Log rank *p* < 0.0001) (Fig. 4). The primary study group consisted of 116 CHP patients. Demographic data and average visual score, CALIPER score and PFT data are provided in Table 1. Interobserver variation values for the visual scores are provided in Additional file 1 (Table 1).

**Table 1:** Patient age, gender and mean and standard deviations of pulmonary function indices, CALIPER and visually scored CT parameters, and echocardiography data in patients with chronic hypersensitivity pneumonitis. Data represent mean values with standard deviations. CT computed tomography, FEV1 forced expiratory volume in one second, FVC forced vital capacity, DLco diffusing capacity for carbon monoxide, Kco carbon monoxide transfer coefficient, TLC total lung capacity, CPI composite physiological index, ILD interstitial lung disease, RVSP right ventricular systolic pressure, TxBx = traction bronchiectasis. Baseline variables in patients with hypersensitivity pneumonitis

Variable (n = 116 unless stated) Units are percentage unless stated	Value
Median Age (years)	58 · 5
Male/female	39/77
Survival (alive/dead)	77/39
Never smokers/ever/current-smokers (*n* = 128)	71/42/2
Pack years	17.5 ± 16.2
Follow up time (months)	58.0 ± 17 · 5
FEV1 % predicted (*n* = 112)	68 · 6 ± 19 · 7
FVC % predicted (*n* = 112)	72 · 2 ± 22 · 8
DLco % predicted (*n* = 109)	42 · 9 ± 15 · 8
Kco % predicted (*n* = 109)	70 · 1 ± 16 · 3
TLC% predicted (*n* = 103)	72 · 0 ± 16 · 8
CPI (*n* = 108)	47 · 9 ± 15 · 2
Echocardiography RVSP (mmHg) (*n* = 58)	35 · 0 ± 14 · 9
CALIPER ILD extent	24 · 3 ± 22 · 5
CALIPER Fibrosis extent	4 · 8 ± 4 · 1
CALIPER Ground glass opacity	19 · 6 ± 20 · 9
CALIPER Reticular pattern	4 · 4 ± 3 · 9
CALIPER Honeycombing	0 · 3 ± 0 · 6
CALIPER Emphysema	0 · 5 ± 1 · 7
CALIPER Pulmonary vessel volume	4 · 5 ± 1 · 9
CALIPER Normal lung	70 · 7 ± 23 · 8
Visual ILD extent	62 · 1 ± 24 · 6
Visual fibrosis extent	27 · 4 ± 19 · 0
Visual Ground glass opacity	33 · 4 ± 24 · 0
Visual Reticular pattern	26 · 4 ± 18 · 2
Visual Honeycombing	0 · 9 ± 4 · 1
Visual Consolidation	0 · 8 ± 3 · 3
Visual Mosaicism	5.0 ± 7 · 1
Visual Emphysema	3 · 0 ± 8v4
Visual TxBx severity (max score 18)	5 · 9 ± 4 · 7
Main pulmonary artery diameter (mm)	29 · 6 ± 4 · 9
Ascending aorta diameter (mm)	33 · 1 ± 3 · 9
Pulmonary artery:ascending aorta ratio	0.9 ± 0 · 1

### CHP mortality analyses

Strong univariate CALIPER and visually-scored CT predictors of mortality in the CHP cohort included reticular pattern, honeycombing, visual traction bronchiectasis, PVV and the fibrosis score (Table 2). CALIPER and visually scored emphysema were not predictive of survival.

**Table 2:** Univariate Cox regression analysis of chronic hypersensitivity pneumonitis patients demonstrating mortality according to CALIPER indices (top white), visually derived CT indices (light grey), pulmonary function tests (dark grey), echocardiography, and clinical indices (lower white). ILD = Interstitial lung disease, DA = decreased attenuation, TxBx = traction bronchiectasis, PA = pulmonary artery, Ao = Aorta, PVV = pulmonary vessel volume, FEV_1_ = forced expiratory volume in one second, FVC = forced vital capacity, TLC = total lung capacity, DLco = diffusing capacity for carbon monoxide, Kco = Carbon monoxide transfer coefficient, CPI = composite physiologic index, RVSP = right ventricular systolic pressure, NS = not significant

	Number of patients	Hazard ratio	*P* value	95.0% Confidence interval	
				Lower	Upper
Caliper indices					
Total ILD extent	116	1 · 02	0 · 001	1 · 01	1 · 03
Total fibrosis extent	116	1 · 32	<0 · 0001	1 · 22	1 · 43
Ground glass opacity	116	1 · 02	0 · 02	1 · 00	1 · 03
Reticular pattern	116	1 · 33	<0 · 0001	1 · 22	1 · 44
Honeycombing	116	2 · 71	0 · 001	1 · 54	4 · 76
Emphysema	116		NS		
Normal lung	116	0 · 98	0 · 001	0 · 97	0 · 99
PVV	116	1 · 56	<0 · 0001	1 · 31	1 · 84
Visual indices					
ILD extent	116		NS		
Fibrosis extent	116	1 · 06	<0 · 0001	1 · 04	1 · 08
Ground glass opacity	116	0 · 98	0 · 02	0 · 97	1 · 00
Reticular pattern	116	1 · 05	<0 · 0001	1 · 03	1 · 07
Honeycombing	116	1 · 19	0 · 001	1 · 07	1 · 31
Consolidation	116		NS		
Total emphysema	116		NS		
Mosaicism	116		NS		
TxBx severity	116	1 · 16	<0 · 0001	1 · 09	1 · 23
Main PA	116	1 · 09	0 · 002	1 · 03	1 · 15
Aorta	116		NS		
PA:Ao ratio	116	1 · 03	0 · 02	1 · 01	1 · 05
Pulmonary function indices					
FEV_1_	112	0 · 98	0 · 02	0 · 96	1 · 00
FVC	112	0 · 98	0 · 006	0 · 97	0 · 99
TLC	103	0 · 96	0 · 0001	0 · 93	0 · 98
DLco	109	0 · 96	0 · 001	0 · 93	0 · 98
Kco	109		NS		
CPI	108	1 · 04	0 · 001	1 · 02	1 · 07
Other indices					
ECHOCARDIOGRAPHY RVSP	58		NS		
Age	116		NS		
Gender	116		NS		
Smoking history	115		NS		

On bivariate analyses, PVV was a stronger predictor of mortality than FVC, DLco, and CPI (Additional file 1 [Table 2]). The results were maintained following adjustment for patient age and gender with no PFTs retaining significance in the model. In a subanalysis of CHP patients with either histopathological confirmation or antibody positivity to precipitating antigens, after adjusting for patient age and gender, PVV (*p* = 0 · 005) remained a stronger predictor of mortality than FVC (*p* = 0 · 10), DLco (*p* = 0 · 009) and CPI (*p* = 0 · 01).

### Benchmarking of an IPF outcome in patients with CHP

**Fig. 5:**
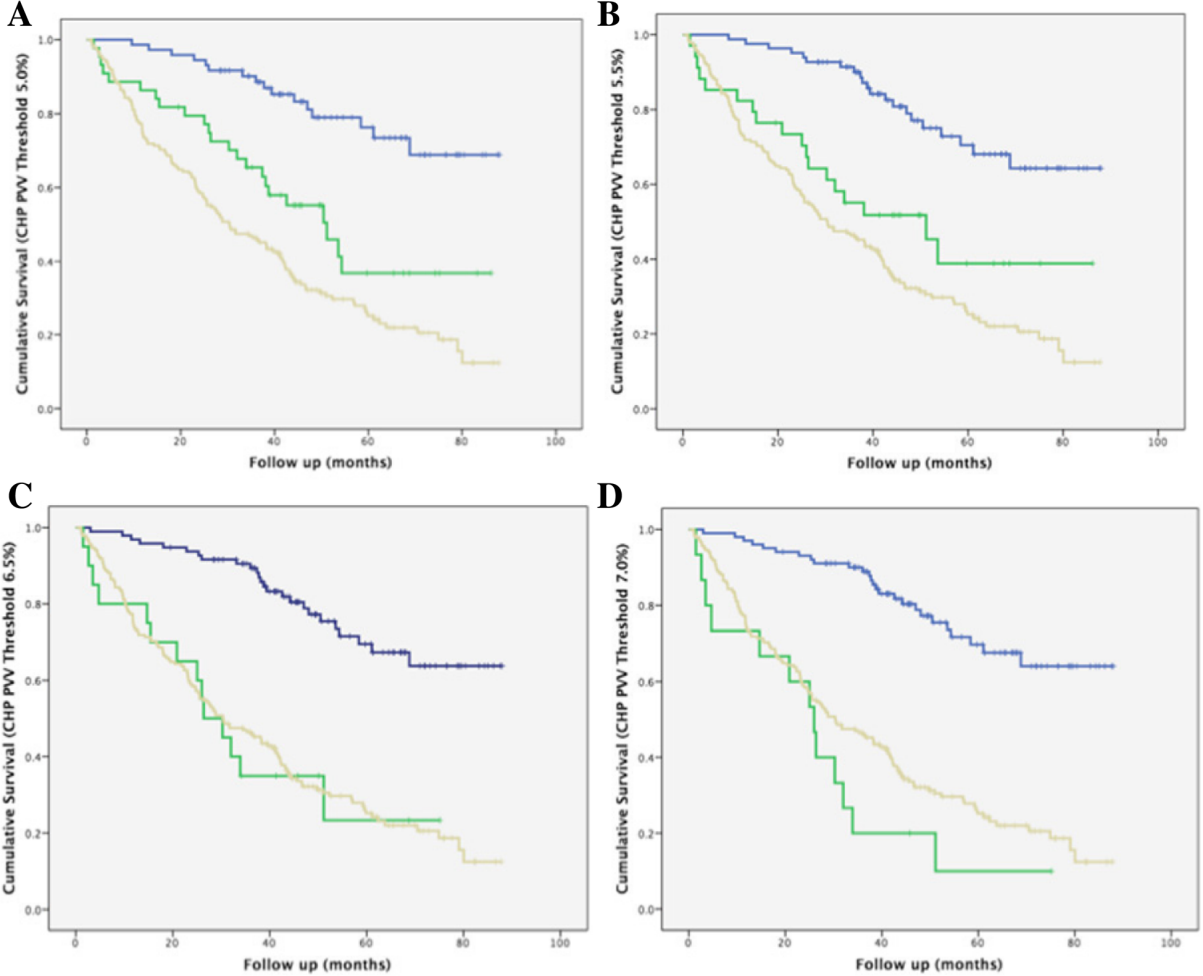
Survival curves for patients with idiopathic pulmonary fibrosis (IPF) (yellow) and chronic hypersensitivity pneumonitis (CHP) (green and blue). CHP cases with end stage disease (denoted by a PaO2 < 7 · 5kPa) were excluded. IPF cases without a measured PaO2 or with end stage disease (denoted by a PaO2 < 7 · 5kPa) were excluded. IPF patients (*n* = 185) were compared to CHP cases at pulmonary vessel volume (PVV) thresholds above (green) and below (blue) 5%, 5 · 5%, 6 · 5% and 7% (**a**–**d**). The IPF and poor-outcome CHP curves were most comparable at a CHP PVV threshold of 6 · 5% where the mean survival in the IPF population (38 · 4 ± 2 · 2 months; *n* = 185) was found to be similar to the poor-outcome CHP population (35 · 3 ± 6 · 1 months; *n* = 20) and distinct from the good-outcome CHP population (71 · 3 ± 2 · 8 months; *n* = 96). The PVV > 6% threshold contained only two patients more than the PVV > 6 · 5% threshold which did not significantly alter the results and accordingly was not been formally plotted

A consecutive population of IPF patients (*n* = 283), presenting to our institution over the same period as the CHP study population was analyzed to establish a mortality profile of IPF. The IPF population has been previously evaluated in two studies [9, 13]. To identify and exclude patients with respiratory failure and end-stage disease, in a similar way to the CHP cohort, all IPF patients without a measured Pa0_2_ (*n* = 70), as well as those patients with a Pa0_2_ < 7 · 5kPa (*n* = 28) were excluded. 185 IPF patients were subsequently evaluated with Kaplan Meier analysis which identified the mean survival to be 38 · 4 ± 2 · 2 months (Fig. 5). Of the final population of 185 IPF patients, 74/185 (40%) patients demonstrated a definite UIP pattern on CT and 102/185 (55%) patients demonstrated a possible UIP pattern on CT. 9/185 (5%) patients had a CT pattern that was inconsistent with a UIP pattern, but which was characterised as UIP on open lung biopsy, and confirmed as IPF following MDT discussion with no support for a diagnosis of CHP or connective tissue disease related-ILD (alternative explanations of UIP).

### Derivation of poor-outcome using PVV thresholds

CHP patients with an IPF-like outcome were identified by separating the CHP population into two age and gender-matched cohorts comprising 58 patients (Additional file 1 [Table 3]). No significant differences were identified for PFTs or CT parenchymal pattern scores between groups. Various PVV thresholds predictive of mortality were investigated. A PVV threshold >6 · 5% of the lung showed a mean survival most comparable to IPF patients in both CHP groups: Group 1 mean survival = 39 · 7 ± 7 · 8 months (*n* = 11; 19%); Group 2 mean survival = 28 · 2 ± 8 · 1 months (*n* = 9; 16%).

**Table 3:** Patient demographics, pulmonary function indices, CALIPER and visually scored CT parameters and echocardiography data in 2 groups of chronic hypersensitivity pneumonitis patients. Group 1 had an IPF-like outcome (*n* = 20) and group 2 had a good outcome (*n* = 96). Differences between groups were assessed using the *T*-test for continuous variables, the Mann Whitney *U* test for differences in continuous median values and the Chi-Squared test for categorical variables. CT = computed tomography, FEV1 = forced expiratory volume in one second, FVC = forced vital capacity, DLco = diffusing capacity for carbon monoxide, Kco = carbon monoxide transfer coefficient, TLC = total lung capacity, CPI = composite physiological index, ILD = interstitial lung disease, RVSP = right ventricular systolic pressure TxBx = traction bronchiectasis, PA = pulmonary artery. Comparison of hypersensitivity pneumonitis patients groups: IPF-like versus non-IPF-like outcome

Variable Units are percentage unless stated	Group 1 (*n* = 20 unless stated in brackets)	Group 2 (*n* = 96 unless stated in brackets)	Group Differences
Median Age (years)	56 · 5	59	NS
Male/female	7/13	32/64	NS
Survival (alive/dead)	6/14	71/25	<0 · 0001
Never/ever/current smokers	15/4/0 (19)	56/38/2	NS
Pack years	28 · 5 ± 26 · 5	16 · 4 ± 14 · 9	NS
FEV1 % predicted	48 · 1 ± 12 · 1 (18)	72 · 5 ± 18 · 4 (94)	<0 · 0001
FVC % predicted	45 · 6 ± 10 · 8 (18)	77 · 3 ± 20 · 9 (94)	<0 · 0001
DLco % predicted	28 · 1 ± 8 · 4 (16)	45 · 5 ± 15 · 4 (93)	<0 · 0001
Kco % predicted	70 · 4 ± 16 · 4 (16)	70 · 1 ± 16 · 3 (93)	NS
TLC% predicted	50 · 9 ± 7 · 2 (14)	75 · 3 ± 15 · 4 (89)	<0 · 0001
CPI	64 · 6 ± 6 · 5 (16)	45 · 0 ± 14 · 3 (92)	<0 · 0001
Echocardiography RVSP (mmHg)	33 · 0 ± 6 · 5 (8)	35 · 4 ± 15 · 9 (50)	NS
CALIPER ILD extent	58 · 6 ± 17 · 2	17 · 2 ± 15 · 9	<0 · 0001
CALIPER Ground glass opacity	48 · 7 ± 21 · 1	13 · 5 ± 14 · 9	<0 · 0001
CALIPER Reticular pattern	9 · 4 ± 5 · 1	3 · 4 ± 2 · 5	<0 · 0001
CALIPER Honeycombing	0 · 5 ± 1 · 1	0 · 3 ± 0 · 4	NS
CALIPER Emphysema	0 · 2 ± 0 · 4	0 · 5 ± 1 · 8	NS
CALIPER Pulmonary vessel volume	7 · 7 ± 0v8	3 · 8 ± 1 · 3	<0 · 0001
Visual ILD extent	74 · 9 ± 17 · 4	59 · 5 ± 25 · 1	=0 · 002
Visual Ground glass opacity	24 · 1 ± 13 · 5	35 · 3 ± 25 · 3	=0.006
Visual Reticular pattern	44 · 4 ± 14 · 7	22 · 7 ± 16 · 6	<0 · 0001
Visual Honeycombing	3 · 6 ± 9 · 5	0 · 4 ± 1 · 1	NS
Visual Emphysema	0 · 3 ± 0 · 5	3 · 5 ± 9 · 2	=0 · 001
Visual TxBx severity (max score 18)	9 · 2 ± 4 · 9	5 · 2 ± 4 · 4	=0 · 0004
Main PA diameter (mm)	31 · 5 ± 4 · 5	29 · 2 ± 4 · 9	NS
Ascending aorta diameter (mm)	32 · 2 ± 4 · 3	33 · 3 ± 3 · 9	NS
PA:ascending aorta ratio	1.0 ± 0.2	0.9 ± 0.1	=0.006

The two CHP groups were combined and PVV thresholds were reanalyzed and compared to outcomes in the IPF population (Fig. 5). No statistically significant difference between Kaplan Meier survival curves was identified on comparison of the IPF and poor-outcome CHP groups when using a PVV threshold of 6 · 5% (Log rank test *p* = 0 · 89).

Percent-predicted DLco and CPI thresholds indicative of an IPF-equivalent outcome were examined in the CHP population (*n* = 116) and compared to the 6 · 5% PVV threshold. A DLco of <25% predicted was associated with a mean survival of 41 · 8 ± 8 · 1 months and a CPI threshold >65 was associated with a mean survival of 38 · 1 ± 6 · 1 months. The PVV threshold was a stronger predictor of mortality than either PFT threshold.

### Combined analysis of IPF and poor-outcome CHP cases

The 20 CHP cases with an IPF-like outcome were combined with the 185 IPF cases and evaluated together using Cox proportional hazards analyses. An MDT diagnosis of CHP was not associated with an improved outcome over patients diagnosed with IPF on univariate analysis. Results were confirmed following correction for age, gender and disease severity (as estimated by the CPI) and bootstrapping of 1000 samples at a confidence interval level of 95%. Results did not change when the 70 IPF patients without a measured Pa0_2._were included in the IPF cohort.

When patients were categorized as non end-stage disease using an FVC ≥ 50% predicted threshold the results were essentially unchanged (see Additional files 1, Additional file 2 and 3).

### Subanalysis of the poor outcome CHP population

No difference in patient age, gender or smoking history was identified between good and bad outcome CHP patients. 13/20 (65%) of poor outcome CHP patients had histopathological or antibody-positive confirmation of a CHP diagnosis and more ILD measured visually (*p* = 0 · 008) and by CALIPER (*p* < 0 · 0001) than good outcome patients (Table 3). Overall, CALIPER variables were better able to identify differences between CHP outcome groups than visual CT scores. The mean FVC, DLco and CPI between groups were significantly different (*p* < 0 · 0001). However no difference was identified in either the mean RVSP (*p* = 0 · 96), or an RVSP value indicative of pulmonary hypertension [>50 mm Hg] [16] between outcome groups. The lack of a vasculopathic signal was reinforced by the lack of an appreciable difference in Kco values between groups (*p* = 0 · 88).

## Discussion

Our study is the first to our knowledge to evaluate the prognostic value of quantitatively derived CT variables in patients with chronic hypersensitivity pneumonitis. Importantly, we have shown that the pulmonary vessel volume (PVV) provides a robust prognostic signal, independent of PFTs and thresholding of the PVV allowed clear prognostic distinctions to be made in CHP patients. 17% of the CHP study population had a PVV above 6 · 5% of the total lung volume, and these patients demonstrated a rate of disease progression, nearly identical to that of IPF.

Previous studies have reported that a proportion of CHP patients exhibit rapid decline and progression to death, similar to patients with IPF [4, 5]. A study of 47 histologically-confirmed UIP patients performed by Pérez-Padilla et al [4] showed that after correcting for CT disease extent, patients exposed to avian antigens had similar outcomes to patients unexposed to avian antigens [4]. A study by Mooney et al [17] demonstrated that the quartile of CHP patients with the most extensive fibrosis (the sum of visual CT reticulation and honeycomb extent scores), had an outcome comparable to IPF at 4 years. However the study by Mooney et al included CHP patients with respiratory failure and end-stage disease who would be expected to have a poor, IPF-like outcome. In our study our specific aim was to demonstrate that even with the exclusion of patients with end-stage disease, a proportion of CHP patients progress to death with an IPF-like disease course.

A prior study in CHP patients with interspaced CT imaging demonstrated that computer-derived CT variables were strongly predictive of mortality [18]. However the interspaced nature of the dataset precluded certain variables such as the pulmonary vessel volume form being adequately examined as a predictor of mortality. Given that a previous report in patients with IPF demonstrated the PVV to be the strongest CT variable for predicting mortality [9], it was a logical step to examine this key variable in a separate CHP population with volumetric CT imaging. The similar severity-adjusted survival between a subgroup of CHP patients and IPF patients identified in the current study corroborates previous observations that a subset of CHP patients have an outcome nearly identical to IPF. [2, 4, 5] Whilst our findings do not identify the mechanism for this difference, three potential contributing factors could be considered.

Firstly, the PVV threshold may identify CHP patients that have more aggressive lung disease. Alternatively PVV may be providing added information regarding pulmonary vasculopathy, though the similarities in RVSP and Kco between good and bad outcome CHP groups makes this unlikely. Lastly vessel size may be a better marker of interstitial severity than functional indices. The high negative intrathoracic pressures generated during respiration in patients with fibrosing lung disease may increase as fibrosis worsens, drawing extra blood into the lungs as well as concomitantly exerting a pull on pulmonary vessel walls. The mortality signal associated with a high PVV may therefore reflect worsening pulmonary fibrosis.

Intriguingly, a study evaluating pulmonary angiogenesis in patients with CHP has identified elevated levels of vascular endothelial growth factor in the lungs of CHP patients when compared to control subjects [19]. Angiogenesis represents a physiological, hypoxia-induced response within the tissues to facilitate improvements in gas exchange. Therefore an increase in the PVV may represent a response to the progressive impairment in gas exchange that results as the interstitial damage progresses.

The PVV threshold of >6 · 5% of the lung demonstrated a stronger distinction between groups than any individual PFT and has the additional benefit of avoiding the issue of observer disagreement associated with subjective visual CT scoring. Furthermore, given recent evidence suggesting variability between MDTs in making a CHP diagnosis [7], we were able to confirm the PVV signal strength in patients with either a histopathological CHP diagnosis or antibody positivity.

Significant univariate predictors of mortality in the current study are in line with previous reports that have identified CT markers of fibrosis including reticulation [17], honeycombing [20, 21], traction bronchiectasis [3] and a fibrosis score (sum of reticulation and honeycombing) [1, 17, 22] as predictors of a poor prognosis in CHP. A mosaic attenuation pattern (scored as the decreased attenuation component), however, was not predictive of mortality in contrast to the findings in histopathologically proven CHP cases of Lima et al. [1] Emphysema extent scored on CT had no bearing on prognosis, a result which could arguably be reconciled with the relatively limited extent of emphysema in our CHP cohort.

In the CHP group with an IPF-like outcome, the majority (79%) of patients were never smokers and unlike previous reports in CHP, smoking was not associated with a worse outcome [23]. Similarly, the presence of pulmonary hypertension was not associated with a worsened prognosis in contrast to the study by Koschel et al. [24] However the study by Koschel et al [24] did not correct for baseline disease extent when analyzing outcome in CHP patients with pulmonary hypertension which could have negated the study findings.

Of the PFTs, the total lung capacity (TLC) was the strongest predictor of mortality on univariate analysis, echoing the findings of Mooney et al [17]. However, the same association of TLC with mortality was not replicated in a study by Vourlekis et al [2]. On multivariate analysis, CT variables, particularly CALIPER PVV, were stronger predictors of mortality than FVC, DLco, and CPI. The findings mirror the results of the study by Walsh et al which reported the superiority of visual CT variables over any PFTs in predicting mortality in CHP [3].

In our analysis, we deliberately excluded patients with end-stage fibrosing lung disease to allow the identification of patients at risk of a poor outcome, but in whom, targeted intervention might reasonably be expected to modify disease progression. Patients with end-stage disease, characterized by the requirement of oxygen therapy at rest, more usually enter palliative care pathways rather than aggressive medical treatment. The poor outcome of such end-stage CHP cases was highlighted in the study by Mooney et al in which, unsurprisingly, oxygen therapy was shown to be predictive of mortality on multivariate analysis [17]. In our study, with the exclusion of end-stage patients, a significant proportion of CHP patients (17%) had an outcome comparable to IPF.

There are limitations to the current study. In an era in which the MDT diagnosis is the accepted diagnostic standard, the number of patients undergoing surgical biopsy for tissue diagnosis is inevitably reduced. Though only 46% of patients in the final study had a surgical biopsy, the diagnosis of CHP on a histopathological examination alone may not be definitive, as studies have shown that the majority of biopsy samples do not show all of the classical pathological features [1, 6]. The PVV variable was found to be liable to minor contamination as areas of reticular pattern, predominantly in the lung periphery, were captured and classified as vessels. The noise associated with the PVV signal when improved in future iterations of similar software would likely strengthen our observations. Though the median age of our CHP population was slightly higher than several prior CHP studies [2, 25, 26], it was similar to the study by Mooney et al [17] whilst the median age of the poor-outcome CHP group was similar to the HP population in the study by Lacasse et al [25]. We do not believe that these limitations diminish the applicability of our results to patients with an MDT diagnosis of CHP.

## Conclusions

In conclusion, we have demonstrated that a significant proportion of CHP patients have an IPF-like outcome and that this can be predicted from the PVV derived from CALIPER software. This novel finding may have a role in identifying the subgroup of CHP patients who may benefit from close monitoring and intervention to control disease progression.

## Additional files

Additional file 1: Brief description of data: Methodological information regarding: pulmonary function test, echocardiography and CT scanning protocols and details regarding CALIPER CT scoring and visual CT scoring and consensus formulation of visual CT scores. A further analysis of end-stage patients defined using an FVC threshold <50% predicted is also included. Two tables highlighting the single determination standard deviation values for the two radiologist scores and a comparison of age and gender matched hypersensitivity pneumonitis groups are also included. (DOCX 125 kb)

Additional file 2: Figure S1. Brief description of data: Survival curves demonstrating outcomes in non end-stage (defined using an FVC threshold >50% predicted) hypersensitivity pneumonitis patients. (PNG 70 kb)

Additional file 3: Figure S2. Brief description of data: Survival curves comparing poor-outcome (defined using an FVC threshold >50% predicted) chronic hypersensitivity pneumonitis patients and idiopathic pulmonary fibrosis patients using Kaplan Meier curve analysis. (PNG 80 kb)

## Abbreviations

Ao: Aorta
BAL: Broncho-alveolar lavage
CALIPER: Computer-aided lung informatics for pathology evaluation and rating
CHP: Chronic hypersensitivity pneumonitis
CI: Confidence interval
CPI: Composite physiologic index
CT: Computed tomography
DA: Decreased attenuation
DLco: Diffusing capacity for carbon monoxide
FEV1: Forced expiratory volume in one second
FVC: Forced vital capacity
GGO: Ground glass opacity
HR: Hazard ratio
ILD: Interstitial lung disease
IPF: Idiopathic pulmonary fibrosis
Kco: Carbon monoxide transfer coefficient
MDT: Multi-disciplinary team
PA: Pulmonary artery
Pa0_2_: Partial pressure of oxygen
PFT: Pulmonary function test
PVV: Pulmonary vessel volume
RVSP: Right ventricular systolic pressure
TLC: Total lung capacity
TxBx: Traction bronchiectasis
UIP: Usual interstitial pneumonia
