# Supplementary material for: Chronic hypersensitivity pneumonitis: identification of key prognostic determinants using automated CT analysis
Source: BMC Pulm Med. 2017 May 4;17:81. doi: 10.1186/s12890-017-0418-2 (PMC5418678; doi:10.1186/s12890-017-0418-2)
Supplement: Supplementary file 1 — Brief description of data: Methodological information regarding: pulmonary function test, echocardiography and CT scanning protocols and details regarding CALIPER CT scoring and visual CT scoring and consensus formulation of visual CT scores. A further analysis of end-stage patients defined using an FVC threshold <50% predicted is also included. Two tables highlighting the single determination standard deviation values for the two radiologist scores and a comparison of age and gender matched hypersensitivity pneumonitis groups are also included. (DOCX 125 kb) [file 12890_2017_418_MOESM1_ESM.docx]

**Supplementary appendix**

**Pulmonary function tests:**

Pulmonary function tests were analyzed if performed within 3 months of the corresponding HRCT scan. Spirometry (Jaeger Master screen PFT, Carefusion Ltd., Warwick, UK), plethysmographic lung volumes (Jaeger Master screen Body, Carefusion Ltd., Warwick, UK), and diffusion capacity for carbon monoxide (DLco) (Jaeger Master screen PFT, Carefusion Ltd., Warwick. UK) were used to measure pulmonary function according to established protocols [^1^](#_ENREF_1).Parameters assessed included: forced expiratory volume in one second (FEV_1_), forced vital capacity (FVC), total lung capacity (TLC), carbon monoxide transfer coefficient (Kco), and single breath carbon monoxide diffusing capacity corrected for hemoglobin concentration (DLco). Pulmonary function tests results were expressed as percent predicted values using the patient’s age, sex, race, and height. The composite physiological index (CPI) was calculated using the formula: 91·0 - (0·65 x % predicted DLco) - (0·53 x % predicted FVC) + (0·34 x % predicted FEV_1_) [^2^](#_ENREF_2).

**Echocardiography**

Right ventricular systolic pressure was calculated using the modified Bernoulli equation and was considered to be equal to the transtricuspid gradient + right atrial pressure, where the transtricuspid gradient = 4V^2^ (where v is equal to the peak velocity of tricuspid regurgitation in meters per second).[^3^](#_ENREF_3) The right atrial pressure was calculated as 5mmHg unless there were indications of functional impairment. A threshold of >50mmHg was considered to represent pulmonary hypertension. [^4^](#_ENREF_4)

**CT protocol**

The CT scans were obtained using a 64-slice multiple detector CT scanner (Somatom Sensation 64, Siemens, Erlangen, Germany) or a 4-slice multiple detector CT scanner (Siemens Volume Zoom, Siemens, Erlangen, Germany. To satisfy requirements for processing by the CALIPER algorithm, all scans were reconstructed using a high spatial frequency, B70 kernel (Siemens, Munich, Germany). All patients were scanned from lung apices to bases, supine, at full inspiration, with 1.0mm section thicknesses using a peak voltage of 120kVp with tube current modulation. Images were viewed at window settings optimized for the assessment of the lung parenchyma (width 1500 H.U.; level -500 H.U.).

**CALIPER CT evaluation**

Data processing: Initial data processing steps involved extraction of the lung from the surrounding thoracic structures and segmentation into upper, middle and lower zones. Lung segmentation was performed with an adaptive density-based morphological approach [^5^](#_ENREF_5) whilst airway segmentation involved iterative three-dimensional region growing, density thresholding (thresholds including -950HU and -960HU) and connected components analysis. Pulmonary vessels were extracted using optimized multi-scale tubular structure enhancement filters [^6^](#_ENREF_6).

Parenchymal tissue type classification was applied to 15x15x15 voxel volume units using texture analysis, computer vision-based image understanding of volumetric histogram signature mapping features and 3D morphology[^7^](#_ENREF_7). The CALIPER tool was trained by sub-specialty thoracic radiologist consensus assessment of pathologically confirmed datasets [^7^](#_ENREF_7)^,^ [^8^](#_ENREF_8).

**Visual CT evaluation**

CTs were visually scored on a lobar basis using a continuous scale. The total interstitial lung disease (ILD) extent was initially estimated to the nearest 5%, then sub-classified into four patterns: reticular pattern, ground glass opacification, honeycombing and consolidation, using definitions from the Fleischner Society glossary of terms for thoracic imaging [^9^](#_ENREF_9). To derive a lobar percentage for each parenchymal pattern, the total lobar ILD extent was multiplied by individual lobar parenchymal pattern extents and divided by 100. Furthermore, the percentage (to the nearest 5%) of each lobe that contained mosaicism (decreased attenuation component) or emphysema was recorded.

The individual lobar percentages of each parenchymal pattern were summed for each radiologist and divided by six to create an averaged lobar score per pattern, per scorer per case.

Traction bronchiectasis, as defined in the Fleischner society glossary of terms [^9^](#_ENREF_9), was assigned with a categorical “severity” score that took into account the average degree of airway dilatation within areas of fibrosis as well as the extent of dilatation throughout the lobe and was given a gestalt score of: none=0, mild=1, moderate=2, severe=3.

An initial training dataset of 15 non-study cases was used to identify pre-existing biases. The scores of the test cases were reviewed by a third radiologist (JJ) and the most widely discrepant results were discussed with the two study scorers prior to the scoring of the study CTs.

The identification of discrepancies and skewing of CT scores between scorers was derived by evaluating distribution curves of the differences between radiologist scores for each parenchymal pattern. The most disparate 5% (two standard deviations) of values had a consensus derived by the two scorers with knowledge of the previous scores. If a single parenchymal subtype extent was changed at consensus, the other parameters were modified, following CT review, to retain an overall 100% for the sum of the four parenchymal subtypes. Similarly, if the lobar percentages of total interstitial disease, emphysema or mosaicism varied, the other two parameter extents were rescored.

**Analysis using an FVC≥50% predicted threshold**

When the same PVV threshold of 6·5% was examined in CHP patients deemed to have end stage disease using an FVC≥50% predicted threshold, the results were similar to those identified with a PaO_2_ threshold ≥7.5kPa. A PVV threshold of 6·5% demonstrated an IPF-like outcome in CHP patients identified as being non end-stage disease using a FVC≥50% predicted threshold: mean survival=37.4±8.3 months (n=11/103; 11%)[Supplementary Figure 1]. Again, when compared to the IPF population with a FVC≥50% predicted threshold, no statistically significant difference between Kaplan Meier survival curves was identified on comparison of the IPF and poor-outcome CHP groups when using a PVV threshold of 6·5% (Log rank test p=0·68)[Supplementary Figure 2].

Single determination standard deviation for visual CT scores

| **Visual CT Variable (n = 129)** |  | **Single determination standard deviation** |
| --- | --- | --- |
| CT Interstitial lung disease extent |  | 11·95 |
| CT Ground glass density |  | 13·53 |
| CT Reticulation |  | 6·33 |
| CT Honeycombing |  | 1·46 |
| CT Consolidation |  | 2·25 |
| CT Total emphysema |  | 3·46 |
| CT Mosaic attenuation |  | 4·13 |
| CT Traction bronchiectasis severity |  | 1·46 |

**Supplementary Table 1**. Single determination standard deviation for visual CT scores. CT=computed tomography.

Multivariate Cox analyses comparing pulmonary vessel volume (PVV) with functional indices as predictors of outcome

|  | Number of patients | Hazard ratio | P Value | 95.0% Confidence Interval | |
| --- | --- | --- | --- | --- | --- |
|  |  |  |  | Lower | Upper |
| CALIPER PVV | 112 | 1.77 | <0.0001 | 1.35 | 2.31 |
| FVC | 112 | 1·02 | 0·21 | 0.99 | 1·04 |
| CALIPER PVV | 109 | 1·40 | 0·004 | 1·12 | 1·75 |
| DLco | 109 | 0.98 | 0·18 | 0.95 | 1·00 |
| CALIPER PVV | 108 | 1·44 | 0·004 | 1·13 | 1·85 |
| CPI | 108 | 1.01 | 0·52 | 0.98 | 1.05 |
| **CALIPER PVV** | 112 | 1.74 | <0.0001 | 1.31 | 2.31 |
| **FVC** | 112 | 1·01 | 0·09 | 0.98 | 1·03 |
| **CALIPER PVV** | 109 | 1·43 | 0·002 | 1·14 | 1·79 |
| DLco | 109 | 0.97 | 0·06 | 0.94 | 1·01 |
| CALIPER PVV | 108 | 1·43 | 0·004 | 1·12 | 1·83 |
| CPI | 108 | 1.02 | 0·19 | 0.99 | 1.06 |

**Supplementary Table 2**. Multivariate Cox analyses comparing pulmonary vessel volume (PVV) with individual functional indices (FVC, DLco, CPI) as predictors of outcome. The analyses were repeated following adjustment for patient age and gender (bold). FVC = forced vital capacity, DLco = diffusing capacity for carbon monoxide, CPI = composite physiological index.

Comparison of age and gender matched hypersensitivity pneumonitis groups

| **Variable**  **Units are percentage unless stated** | **Group1**  **(n=58 unless stated in brackets)** | **Group 2**  **(n=58 unless stated in brackets)** |
| --- | --- | --- |
| Median Age (years) | 58·5 | 58·5 |
| Male/female | 19/39 | 20/38 |
| Survival (alive/dead) | 41/17 | 36/22 |
| Never/ever/current smokers | 31/26/1 | 40/16/1 |
| Pack years | 16·1 ± 16·1 | 19·9 ± 16.7 |
| Follow up | 49·9 ± 20·3 | 44·6 ± 21.7 |
| FEV1 % predicted | 71·3 ± 19·1 (57) | 65·8 ± 20·0 (55) |
| FVC % predicted | 74·7 ± 22·7 (57) | 69·6 ± 22·8 (55) |
| DLco % predicted | 42·2 ±14·4 (56) | 43·7 ± 17·3 (53) |
| Kco % predicted | 68·9 ± 15·2 (56) | 71·5 ± 17·3 (53) |
| TLC% predicted | 73·6 ± 17·3 (53) | 70·2 ± 16·2 (50) |
| CPI | 48·0 ± 14·5 (56) | 47·7 ± 16·0 (52) |
| Echocardiography RVSP (mmHg) | 38·6 ± 18·9 (21) | 33·0 ± 12·1 (37) |
| CALIPER ILD extent | 24·4 ± 24·7 | 24·3 ± 20·2 |
| CALIPER Ground glass opacity | 19·9 ± 23·1 | 19·2 ± 18·6 |
| CALIPER Reticular pattern | 4·2 ± 3·9 | 4·7 ± 3·8 |
| CALIPER Honeycombing | 0·3 ± 0·4 | 0·3 ± 0·7 |
| CALIPER Emphysema | 0·7 ± 2·3 | 0·2 ± 0·5 |
| CALIPER Pulmonary vessel volume | 4·5 ± 2·1 | 4·5 ± 1·7 |
| Visual ILD extent | 58·0 ± 25·0 | 66·2 ± 23·7 |
| Visual Ground glass opacity | 31·0 ± 23·4 | 35·7 ± 24·5 |
| Visual Reticular pattern | 24·6 ± 18·3 | 28·3 ± 18·1 |
| Visual Honeycombing | 0·7 ± 2·1 | 1·2 ± 5·5 |
| Visual Emphysema | 4·1 ± 11·0 | 1·8 ± 4·4 |
| Visual TxBx severity (max score 18) | 5·5 ± 4·7 | 6·3 ± 4·7 |
| Main PA diameter (mm) | 29·7 ± 4·8 | 29·4 ± 5·0 |
| Ascending aorta diameter (mm) | 32·9 ± 4·3 | 33·3 ± 3·6 |

**Supplementary Table 3**. Patient demographics, pulmonary function indices, CALIPER and visually scored CT parameters, and echocardiography data in patients with chronic hypersensitivity pneumonitis matched for age and gender. No differences were identified between groups using the T-test for continuous variables, the Mann Whitney U test for differences in continuous median values and the Chi-Squared test for categorical variables. CT = computed tomography, FEV1 = forced expiratory volume in one second, FVC = forced vital capacity, DLco = diffusing capacity for carbon monoxide, Kco = carbon monoxide transfer coefficient, TLC = total lung capacity, CPI = composite physiological index, ILD = interstitial lung disease, RVSP = right ventricular systolic pressure, TxBx = traction bronchiectasis, PA=pulmonary artery.

Supplementary Figure 1. Chronic hypersensitivity pneumonitis patients were selected as non end-stage disease using a forced vital capacity (FVC) ≥50% predicted threshold (n=103). When examined using a pulmonary vessel volume (PVV) threshold of 6·5%, there was good separation of the population into two groups with differing outcome. Mean survival in patients with a PVV<6.5% (blue)[=92; mean survival=68.6 ± 3·1 months]; mean survival in patients with a PVV>6.5% (green)[n=11; mean survival=37.4 ± 8.3 months]. Log Rank test p=0.01.

Supplementary Figure 2. Chronic hypersensitivity pneumonitis patients (green)[n=11] were compared to idiopathic pulmonary fibrosis patients (blue)[n=244] using Kaplan Meier curve analysis. The idiopathic pulmonary fibrosis and poor-outcome chronic hypersensitivity pneumonitis curves were most comparable at a pulmonary vessel volume threshold of 6·5% where the mean survival in the idiopathic pulmonary fibrosis population (38·6 ± 1·9 months) was found to be similar to the poor-outcome chronic hypersensitivity pneumonitis population (37·4 ± 8·3 months).

**REFERENCES**

1. Quanjer PH. Standardized lung function testing. *Eur Respir J - Suppl*. 1993;**6**:1-100.

2. Wells AU, Desai SR, Rubens MB, et al. Idiopathic pulmonary fibrosis: a composite physiologic index derived from disease extent observed by computed tomography. *Am J Respir Crit Care Med*. 2003;**167**:962-9.

3. Yock PG, Popp RL. Noninvasive estimation of right ventricular systolic pressure by Doppler ultrasound in patients with tricuspid regurgitation. *Circulation*. 1984;**70**(4):657-62.

4. Bossone E, D’Andrea A, D’Alto M, et al. Echocardiography in pulmonary arterial hypertension: from diagnosis to prognosis. *J Am Soc Echocardiogr*. 2013;**26**(1):1-14.

5. Hu S, Hoffman EA, Reinhardt JM. Automatic lung segmentation for accurate quantitation of volumetric X-ray CT images. *IEEE Trans Med Imaging*. 2001;**20**(6):490-8.

6. Shikata H, McLennan G, Hoffman EA, et al. Segmentation of pulmonary vascular trees from thoracic 3D CT images. *Int J Biomed Imaging*. 2009:11.

7. Bartholmai BJ, Raghunath S, Karwoski RA, et al. Quantitative CT imaging of interstitial lung diseases. *J Thorac Imaging*. 2013;**28**(5):298-307.

8. Maldonado F, Moua T, Rajagopalan S, et al. Automated quantification of radiological patterns predicts survival in idiopathic pulmonary fibrosis. *Eur Respir J*. 2014;**43**(1):204-12.

9. Hansell DM, Bankier AA, MacMahon H, et al. Fleischner Society: glossary of terms for thoracic imaging. *Radiology*. 2008;**246**(3):697-722.
