# Supplementary figures and images for: Chronic hypersensitivity pneumonitis: identification of key prognostic determinants using automated CT analysis
Source: BMC Pulm Med. 2017 May 4;17:81. doi: 10.1186/s12890-017-0418-2 (PMC5418678; doi:10.1186/s12890-017-0418-2)

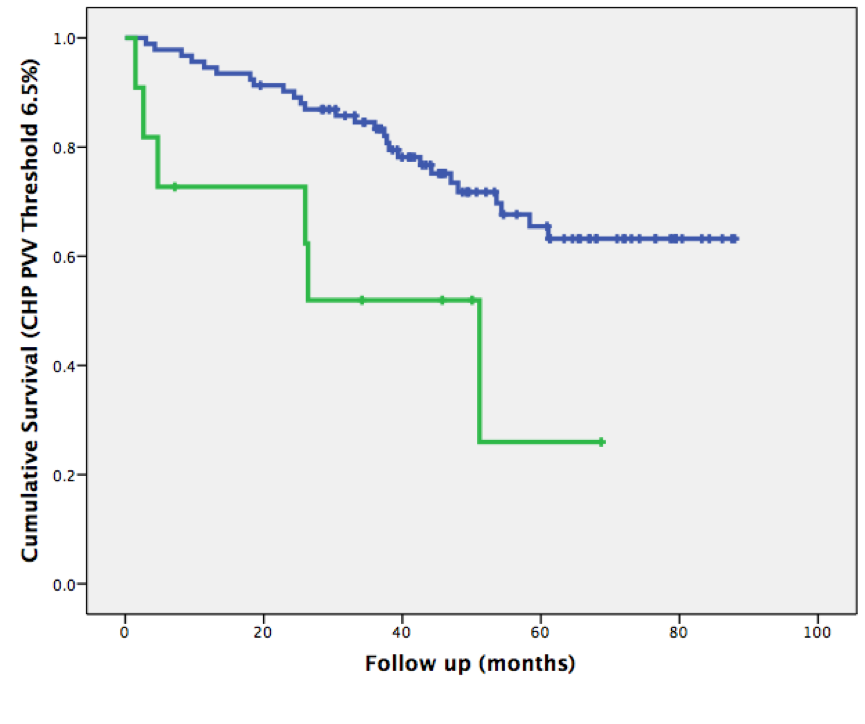

Supplement: Supplementary file 2 — Brief description of data: Survival curves demonstrating outcomes in non end-stage (defined using an FVC threshold >50% predicted) hypersensitivity pneumonitis patients. (PNG 70 kb) [file 12890_2017_418_MOESM2_ESM.png]

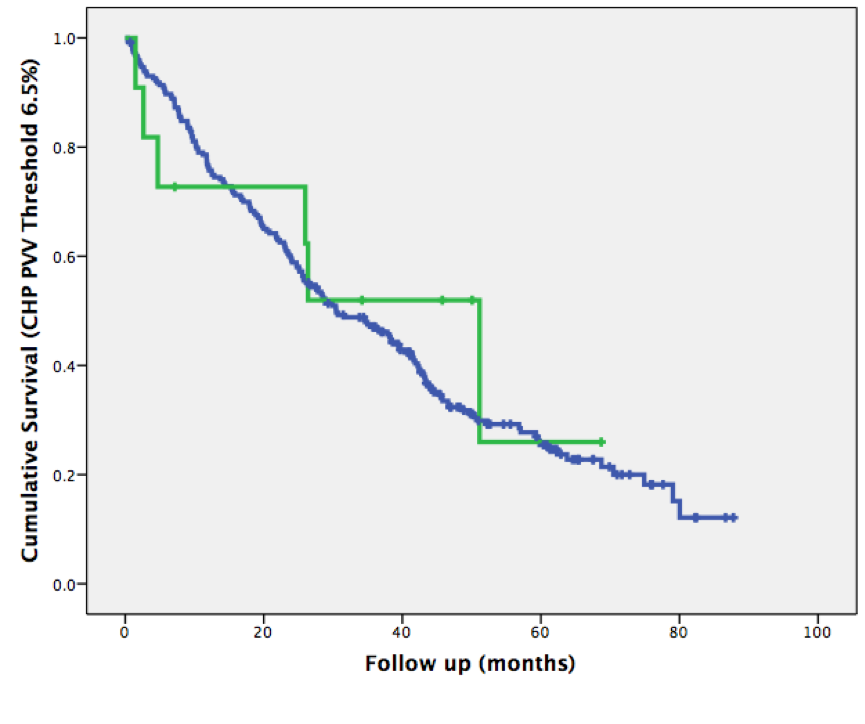

Supplement: Supplementary file 3 — Brief description of data: Survival curves comparing poor-outcome (defined using an FVC threshold >50% predicted) chronic hypersensitivity pneumonitis patients and idiopathic pulmonary fibrosis patients using Kaplan Meier curve analysis. (PNG 80 kb) [file 12890_2017_418_MOESM3_ESM.png]
